# Effect of JAK Inhibitors on Release of CXCL9, CXCL10 and CXCL11 from Human Airway Epithelial Cells

**DOI:** 10.1371/journal.pone.0128757

**Published:** 2015-06-19

**Authors:** Peter S. Fenwick, Patricia Macedo, Iain C. Kilty, Peter J. Barnes, Louise E. Donnelly

**Affiliations:** 1 Airway Disease, National Heart and Lung Institute, Imperial College London, London, United Kingdom; 2 Pfizer Inc, Cambridge, Massachusetts, United States of America; University of Leuven, Rega Institute, BELGIUM

## Abstract

**Background:**

CD8^+^ T-cells are located in the small airways of COPD patients and may contribute to pathophysiology. CD8^+^ cells express the chemokine receptor, CXCR3 that binds CXCL9, CXCL10 and CXCL11, which are elevated in the airways of COPD patients. These chemokines are released from airway epithelial cells via activation of receptor associated Janus kinases (JAK). This study compared the efficacy of two structurally dissimilar pan-JAK inhibitors, PF956980 and PF1367550, and the glucocorticosteroid dexamethasone, in BEAS-2B and human primary airway epithelial cells from COPD patients and control subjects.

**Methods:**

Cells were stimulated with either IFNγ alone or with TNFα, and release of CXCL9, CXCL10 and CXCL11 measured by ELISA and expression of *CXCL9*, *CXCL10* and *CXCL11* by qPCR. Activation of JAK signalling was assessed by STAT1 phosphorylation and DNA binding.

**Results:**

There were no differences in the levels of release of CXCL9, CXCL10 and CXCL11 from primary airway epithelial cells from any of the subjects or following stimulation with either IFNγ alone or with TNFα. Dexamethasone did not inhibit CXCR3 chemokine release from stimulated BEAS-2B or primary airway epithelial cells. However, both JAK inhibitors suppressed this response with PF1367550 being ~50-65-fold more potent than PF956980. The response of cells from COPD patients did not differ from controls with similar responses regardless of whether inhibitors were added prophylactically or concomitant with stimuli. These effects were mediated by JAK inhibition as both compounds suppressed STAT1 phosphorylation and DNA-binding of STAT1 and gene transcription.

**Conclusions:**

These data suggest that the novel JAK inhibitor, PF1367550, is more potent than PF956980 and that JAK pathway inhibition in airway epithelium could provide an alternative anti-inflammatory approach for glucocorticosteroid-resistant diseases including COPD.

## Introduction

Type-1 helper (Th1) lymphocytes and CD8^+^ T cells are elevated in a number of inflammatory diseases including chronic obstructive pulmonary disease (COPD) [1] where these cells are located at the sites of airways obstruction [2, 3] and may contribute to emphysema via the production of granzyme B and perforins [4]. Recently, these cells have been shown to exhibit reduced apoptosis in COPD patients [5] leading to the persistence of these inflammatory cells in the airways. COPD is currently the fifth leading cause of death globally [6] and is increasing in prevalence with estimates that it affects ~10% of the population over the age of 40 [7]. Although inflammation underpins the pathophysiology of COPD, current anti-inflammatory treatments, including glucocorticosteroids, are ineffective [8]. Therefore, alternative strategies are required, for example, reducing recruitment of CD8^+^ cells to the airways of patients with COPD might prove to be beneficial.

The chemokine receptor, CXCR3 is highly expressed by activated Th1 and CD8^+^ lymphocytes and is thought to be involved in recruitment of these cells to the sites of inflammation [9]. CXCR3 binds to three distinct ELR negative ligands, CXCL9 (monokine induced by interferon γ (IFNγ); MIG), CXCL10 (interferon inducible protein of 10 kDa; IP10) and CXCL11 (interferon inducible T-cell α chemoattractant; ITAC) [10], all of which are elevated in the airways of patients with COPD [11] with CXCL10 being elevated in both sputum and serum during a viral exacerbation [12, 13] Although all three of these chemokines bind to the CXCR3 receptor, however CXCL11 has increased affinity and CXCL9 the least, implying a hierarchy of activity [9]. The source of these chemokines in the airways of COPD is unclear, however bronchial airway epithelial cells [14–16] and airway smooth muscle cells [17] release these chemokines following stimulation with interferon (IFN)-γ in both the presence and absence of tumour necrosis factor (TNF)α. Classically, binding of IFNγ activates Janus kinases (JAK) 1 and 2 leading to phosphorylation of signal transducer and activation of transcription (STAT)-1 protein, which subsequently dimerizes and binds to genes containing γ-activated sequences [18] including CXCL9, CXCL10 and CXCL11. STAT-1 independent mechanisms may also be invoked and STAT-3 and STAT-5 have been reported to be activated through the IFNγ receptor [19, 20]. Release of CXCL9, CXCL10 and CXCL11 from both airway epithelial cells and airway smooth muscles can be potentiated by the synergistic interactions of TNFα with IFNγ [14, 21]. In the airways of COPD patients, the concentrations of TNFα are elevated [22] and thus the expression of CXCL9, CXCL10 and CXCL11 by structural cells of the airways is likely to be enhanced, driving lymphocyte recruitment.

Previously, we have shown that the epithelial cell line BEAS-2B releases CXCL9, CXCL10 and CXCL11 in response to IFNγ in a manner that is glucocorticosteroid-insensitive but responsive to inhibition via the IκB kinase, IKK2 [15]. The present study utilized our previous model to assess whether direct inhibition of the JAK pathway could suppress release of CXCL9, CXCL10 and CXCL11 from human lung epithelial cells using two structurally dissimilar compounds. PF956980 ([23]-pyrrolidin-1-yl-methanone hydrate) is a pan-JAK inhibitor with little effect against other kinases, including IKK [24] whereas the novel compound, PF1367550 (4-(3-(1H-benzo[d]imidazol-2-yl)-1H-indazol-6-yl)-3-ethylphenol), is a structurally differentiated, indazole pan-JAK inhibitor with excellent selectivity over non-JAK kinases [25]. Both of these JAK inhibitors were assessed for efficacy and potency to suppress CXCR3 chemokine release from both BEAS-2B cells and human primary airway epithelial cells with a view to understanding the utility of JAK inhibitors as therapeutic agents for inflammation in diseases such as COPD.

## Materials and Methods

### Materials

All chemicals and reagents were from Sigma Chemical Co (Poole, Dorset, UK) unless indicated otherwise. PF956980 and PF1367550 were kind gifts from Pfizer Inc. (Cambridge, Mass). All drugs were reconstituted at a concentration of 10mM in DMSO and diluted from these stock solutions for use in experiments. All experiments were performed with an appropriate vehicle control which had no effect on any of the responses measured.

### Cell culture

BEAS-2B cells (catalogue number CRL-9609) were purchased directly from American Type Culture Collection (Rockville, MD). Keratinocyte serum-free medium (K-SFM), bovine pituitary extract (BPE), and recombinant human epidermal growth factor (EGF) were purchased from Invitrogen (Paisley, UK). BEAS-2B cells were cultured in K-SFM containing 50 μg/ml BPE and 5 ng/ml EGF at 37°C in a humidified atmosphere comprising 5% (v/v) CO_2_ in air. Cells were cultured as monolayers in growth factor-free media for 24 h prior to stimulation. Human primary airway epithelial cells were cultured as monolayers from bronchial brushings from both male and female subjects (Table 1) and cultured in LHC-9 media (Invitrogen, Paisley, UK) in collagen (1% w/v) coated flasks. Samples were obtained from non-smokers, smokers and patients with COPD. Smokers had a smoking history of at least 10 pack-years and COPD patients were stable and fulfilled the American Thoracic Society criteria [26]. The subjects were matched for age and smokers and COPD patients for smoking history (Table 1). All subjects gave informed written consent and the study was approved by the NRES London-Chelsea Research Ethics committee, study number 09/H0801/85.

**Table 1:** Demographic data for subjects providing primary human airway epithelial cells.

	Non-smokersn = 4	Smokersn = 5	COPDn = 7
**Age (years)**	61 ± 10	53 ± 10	66 ± 4
**Gender M:F**	2:2	2:3	2:6
**FEV**_**1**_ **(L)**	2.2 ± 0.4	2.3 ± 0.3	0.8± 0.2** ^##^
**FVC (L)**	2.8 ± 0.6	2.9 ± 0.3	1.8 ± 0.2*
**FEV**_**1**_ **(% predicted)**	79 ± 2	89 ± 8	38 ± 10**
**FVC (% predicted)**	83 ± 1	93± 8	65 ± 9
**FEV**_**1**_ **:FVC**	0.83 ± 0.03	0.76 ± 0.05	0.44 ± 0.09* ^#^
**Smoking History (Pack Years**)	-	23 ± 5	38 ± 6

Data are presented as mean ± S.E.M.

*p<0.05 and

**p<0.01 *vs* smokers

^#^p<0.05 and

##p<0.01 *vs* non-smokers.

### Measurement of CXCL9, CXCL10 and CXCL11

Cell-free supernatants were removed 20h post-stimulation and assayed for CXCL9, CXCL10 and CXCL11 using Duoset ELISA kits (R & D Systems Europe, Abingdon, UK) according to the manufacturer’s instructions. The detection limits of these assays are 62pg/ml, 31pg/ml, and 7.8 pg/ml respectively.

### Cell viability

*C*ell viability was determined colorimetrically by measuring the reduction of 3-[4,5-dimethylthiazol-2-yl]-2,5-diphenyltetrazolium bromide, MTT, to formazan by mitochondrial dehydrogenases, as described previously [27].

### Western Blotting

Enhanced chemiluminescence (ECL) reagent and Hybond-ECL nitrocellulose were obtained from GE Healthcare (Little Chalfont, UK). Bis-Tris SDS-PAGE (4–12%) gels and buffers were purchased from Invitrogen Ltd (Paisley, Scotland, UK). Antibodies against phosphorylated and total STAT-1 were purchased from New England Biolabs (Hitchin, UK). Epithelial cells were treated as indicated in six-well plates and lysed in 10 mM Tris-HCl buffer (pH 7.5) containing 150 mM NaCl, 2 mM EDTA, 1 mM sodium orthovanadate, 1% (v/v) Triton X-100, 1 mM phenylmethylsulfonyl fluoride, 10 μg/ml leupeptin, and 10 μg/ml aprotinin) for 30 min on ice. The lysates were then centrifuged at 12,000*g* for 15 min and protein concentration determined using the BCA protein assay (BioRad, Hemel Hempstead, UK). The proteins within the lysates (20 μg) were resolved with 4–12% Bis-Tris gels and transferred onto nitrocellulose membranes. The membranes were then blocked with 5% (w/v) non-fat milk in TBS containing 0.1% (v/v) Tween 20 for 1 h at room temperature and then incubated with antibodies specific for pSTAT-1 overnight at 4°C. The primary antibody was detected with peroxidase-conjugated secondary antibodies and labelled proteins were detected by ECL.

### STAT Activation Assays (TransAM)

STAT phosphorylation, nuclear translocation and DNA binding was determined using TransAM assays (Actif Motif, Rixensart, Belgium). Cells were treated with JAK inhibitors for 30 min prior to stimulation for 1h. Nuclear extracts were prepared using the Actif Motif assay reagents. Briefly, the nuclear extracts (10 μg) were incubated in DNA coated plates for 1h. After washing, antibodies supplied with the TransAM kit that were specific for the active forms of STAT1, STAT3, STAT5A and STAT5B were added to the wells and incubated for a further hour. The plates were washed and a secondary HRP-labelled anti-goat Ig antibody was added and the plates incubated for an hour. After washing, the assay was developed using the TMB substrate provided and the reaction stopped using 2N sulphuric acid. The plates were read at 450nm with a reference filter of 610nm. Data were then normalised to non-stimulated cells and calculated as fold change from baseline.

### Measurement of mRNA Expression of *CXCL9*, *CXCL10* and *CXCL11*

Total RNA was extracted from cells and reverse transcribed, as described previously [15]. Gene expression was determined by Taqman real-time PCR on a 7500 Real Time PCR system (Applied Biosystems, Life Technologies Ltd, Paisley, UK) using PCR Master Mix Reagent and ‘assays on demand’ (Applied Biosystems). HPRT1 gene expression was used as the housekeeping gene and data presented as δδCT relative to baseline.

### Statistical Analyses

Data are presented as mean ± SEM of ‘*n*’ independent determinations. Concentration-response curves were analysed by least-squares, non-linear iterative regression with the ‘Graphpad Prism’ curve fitting program (GraphPad software, San Diego, USA) and IC_50_ values were subsequently interpolated from curves of best-fit. Statistical differences were determined using the Kruskal–Wallis test followed by Dunn's multiple comparison test or a Wilcoxon signed rank test as appropriate. Values of P<0.05 were considered significant.

## Results

### CXCR3 chemokine release from epithelial cells

**Fig 1:**
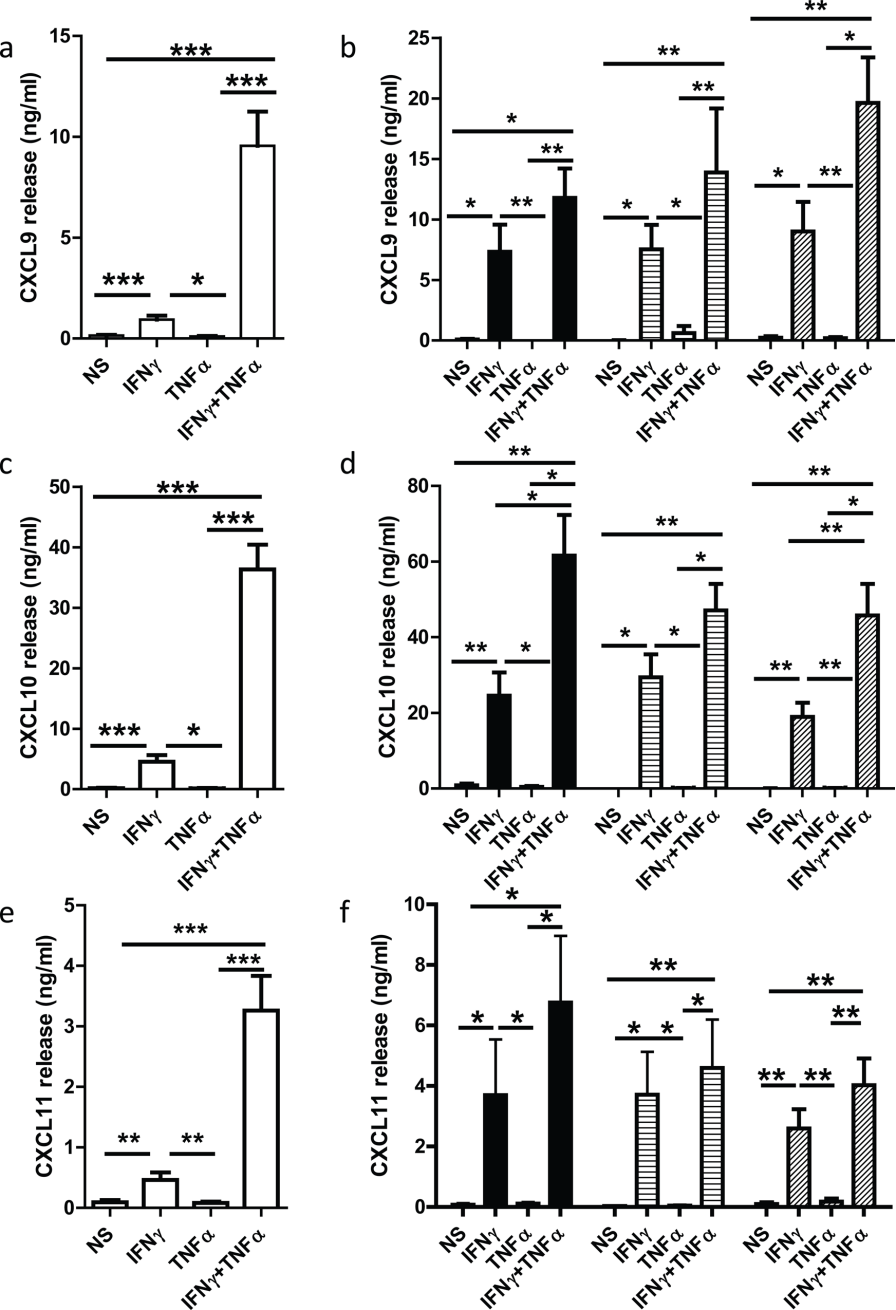
Effect of IFNγ in the presence and absence of TNFα on the release of CXCL9, CXCL10 and CXCL11 from BEAS-2B and primary human airway epithelial cells. BEAS-2B cells, n = 11 (open bars, panels a, c and e) or human primary airway epithelial cells from non-smokers (n = 4) (closed bars), smokers (n = 5) (horizontal lines) or patients with COPD (n = 7) (hatched bars) (panels b, d and f) were incubated for 20h in the absence or presence of IFNγ (10ng/ml) or IFNγ + TNFα (10ng/ml). Media was harvested and the concentrations of CXCL9 (panels a and b), CXCL10 (panels c and d) and CXCL11 (panels e and f) were measured by ELISA. Data are presented as mean ± SEM, where * represents p<0.05, **p<0.01 and ***p<0.001.

Initial experiments were devised to compare the release of CXCL9, CXCL10 and CXCL11 from BEAS-2B cells and primary human airway epithelial cells. IFNγ (10ng/ml) in both the absence and presence of TNFα stimulated the release of CXCL9, CXCL10 and CXCL11 from both cell types (Fig 1). IFNγ and TNFα operated synergistically to stimulate release of CXCL9, CXCL10 and CXCL11 from BEAS-2B cells and (Fig 1) primary human airway epithelial cells with no differences in responses of the two cell types (Fig 1). There were no differences in the responses of human primary airway epithelial cells derived from non-smokers, smokers or patients with COPD with respect to any of the stimuli examined (Fig 1B, 1D and 1F) These data suggest that BEAS-2B cells are a good model for the release of CXCL9, CXCL10 and CXCL11 from human airway epithelia and that human primary airway epithelial cells from COPD patients do not release greater levels of CXCL9, CXCL10 and CXCL11 than cells obtained from control subjects.

### Effect of JAK inhibitors on CXCR3 chemokine release

**Fig 2:**
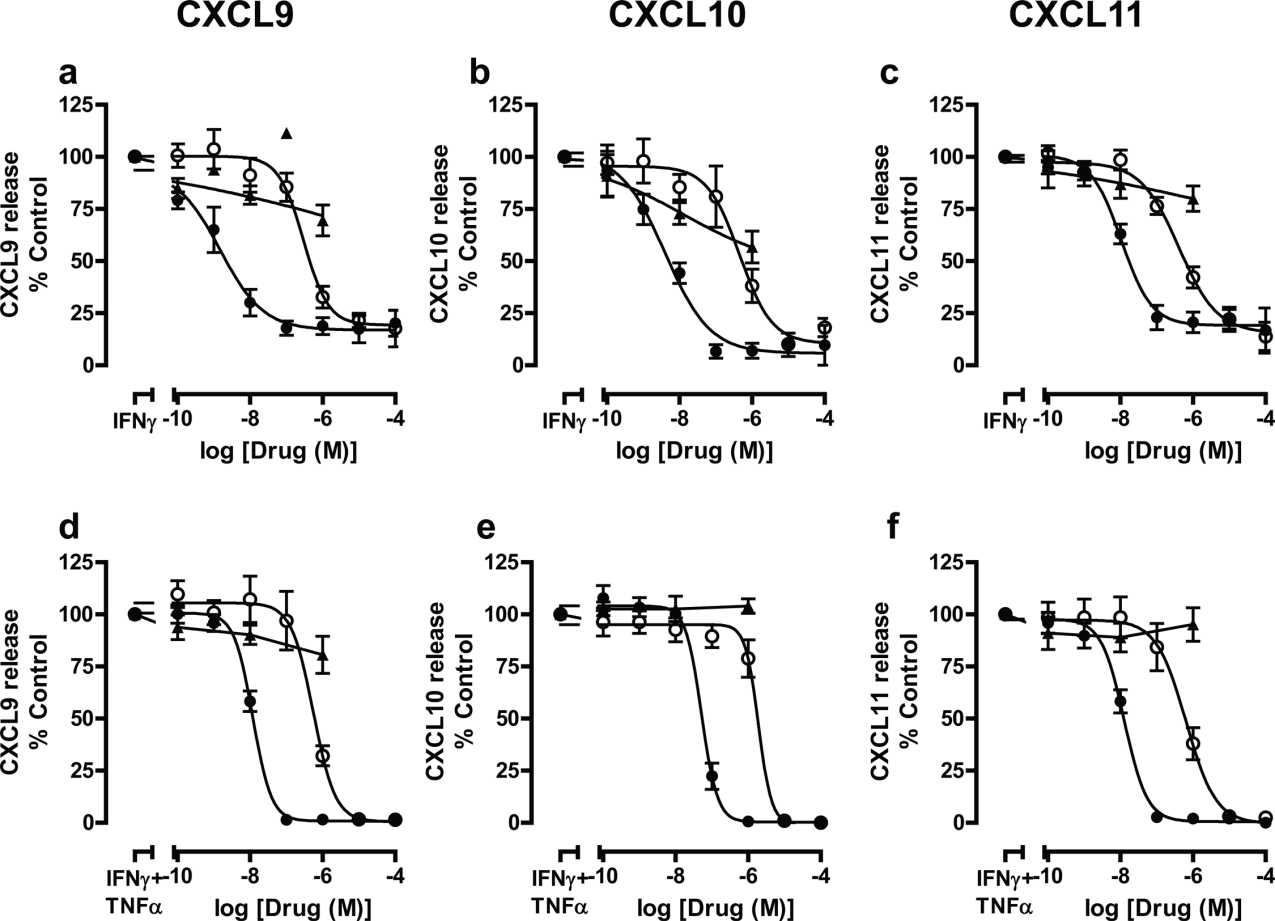
Effect of JAK inhibitors, PF956980 and PF1367550 and dexamethasone on IFNγ and IFNγ + TNFα-stimulated release of CXCL9, CXCL10 and CXCL11 from BEAS-2B cells. BEAS-2B cells were pre-treated and incubated for 1h with either PF956980 (○), PF1367550 (●), or dexamethasone (▲) followed by stimulation for 20h with IFNγ (10ng/ml) (panels a-c) or IFNγ + TNFα (10ng/ml) (panels d-f). Media was harvested and the concentrations of CXCL9 (panels a and d), CXCL10 (panels b and e) and CXCL11 (panels c and f) were measured by ELISA. Data are presented as mean±SEM, n = 7–8.

The effect of two, distinct JAK inhibitors on the release of CXCL9, CXCL10 and CXCL11 was investigated using both BEAS-2B cells and primary airway epithelial cells and compared to the effect of a glucocorticosteroid, dexamethasone. The selectivity of PF956980 has been reported previously [24] with a reported IC_50_ of 22nM for JAK/JAK3 and 188 nM for JAK2 in a cellular assay, whereas the selectivity of PF1367550 is reported in Table 2. PF956980 has been reported to be inactive or with an IC_50_ >30μM against AKT, AuroraA, cdk2, cdk6,CHK1, FGFR1, GSK3b, IKKb, IKKi, INSR, MAPK1, MAPKAP-K2, MASK,MET, PAK4, PDK1, PKCb, ROCK1, TaoK3, TrkA [24]. Both JAK inhibitors, PF956980 and PF1367550, attenuated IFNγ-stimulated and IFNγ+TNFα-stimulated CXCR3 chemokine release from BEAS-2B cells in a concentration-dependent manner (Fig 2) (Table 3), with PF1367550 being ~50-fold more potent than PF956980. This contrasts with dexamethasone, which had a very limited effect on CXCL9 and CXCL11 release (Fig 2A, 2C, 2D and 2F) but a maximal ~50% inhibitory effect on IFNγ-stimulated release of CXCL10 (Fig 2B) but none when CXCL10 was measured following stimulation with IFNγ and TNFα together (Fig 2E).

**Table 2:** Selectivity of PF1367550.

Kinase	IC_50_
JAK1 Km	0.8 nM
JAK1 1 mM	0.4 nM
JAK2 Km	1.0 nM
JAK2 1 mM	1.3 nM
JAK3 Km	1.0 nM
JAK3 1 mM	14 nM
TYK2 Km	0.8 nM
TYK2 1 mM	8.4 nM
IL-2-induced P-STAT5 human T lymphocytes (JAK1/3)	11 nM
IL-2-induced IFNγ release by human T lymphocytes (JAK1/3)	14 nM
IL-12-induced IFNγ release by human T lymphocytes (JAK1/2)	45 nM
MLK1 Km	54 nM
MAP4K4 Km	60 nM
TrkA Km	227 nM
GSK3β Km	617 nM
Protein kinase C β2 Km	665 nM
MST4 Km	692 nM
TAOK2 Km	781 nM

Calculated IC_50_ values for PF1367550 against >50 kinases. Data are presented for the kinases that showed >50% inhibition at 1mM ATP or at the Km as indicated (Data from Coe *et al*., [25]).

**Table 3:** IC_50_ values for PF956980 and PF1367550 on IFNγ and IFNγ+TNFα-stimulated release of CXCL9, CXCL10 and CXCL11 from BEAS-2B cells.

	PF956980 IC_50_ (μM)		PF1367550 IC_50_ (nM)	
Chemokine	IFNγ	IFNγ+TNFα	IFNγ	IFNγ+TNFα
**CXCL9**	0.4 ± 0.1	0.6 ± 0.1	3.6 ± 1.4	13.3 ± 2.0
**CXCL10**	0.6 ± 0.3	2.2 ± 0.3	6.1 ± 2.0	50.3 ± 8.5
**CXCL11**	0.5 ± 0.1	0.9 ± 0. 3	11.5 ± 2.0	13.9 ± 2.6

BEAS-2B cells were pre-treated for 1h in the presence of either PF956980 or PF1367550 prior to stimulation for 20h in the absence or presence of IFNγ (10ng/ml) or IFNγ+TNFα (10ng/ml). After this time, media was harvested and the concentrations of CXCL9, CXCL10 and CXCL11 measured by ELISA. Data are presented as mean±SEM, n = 7–8.

**Fig 3:**
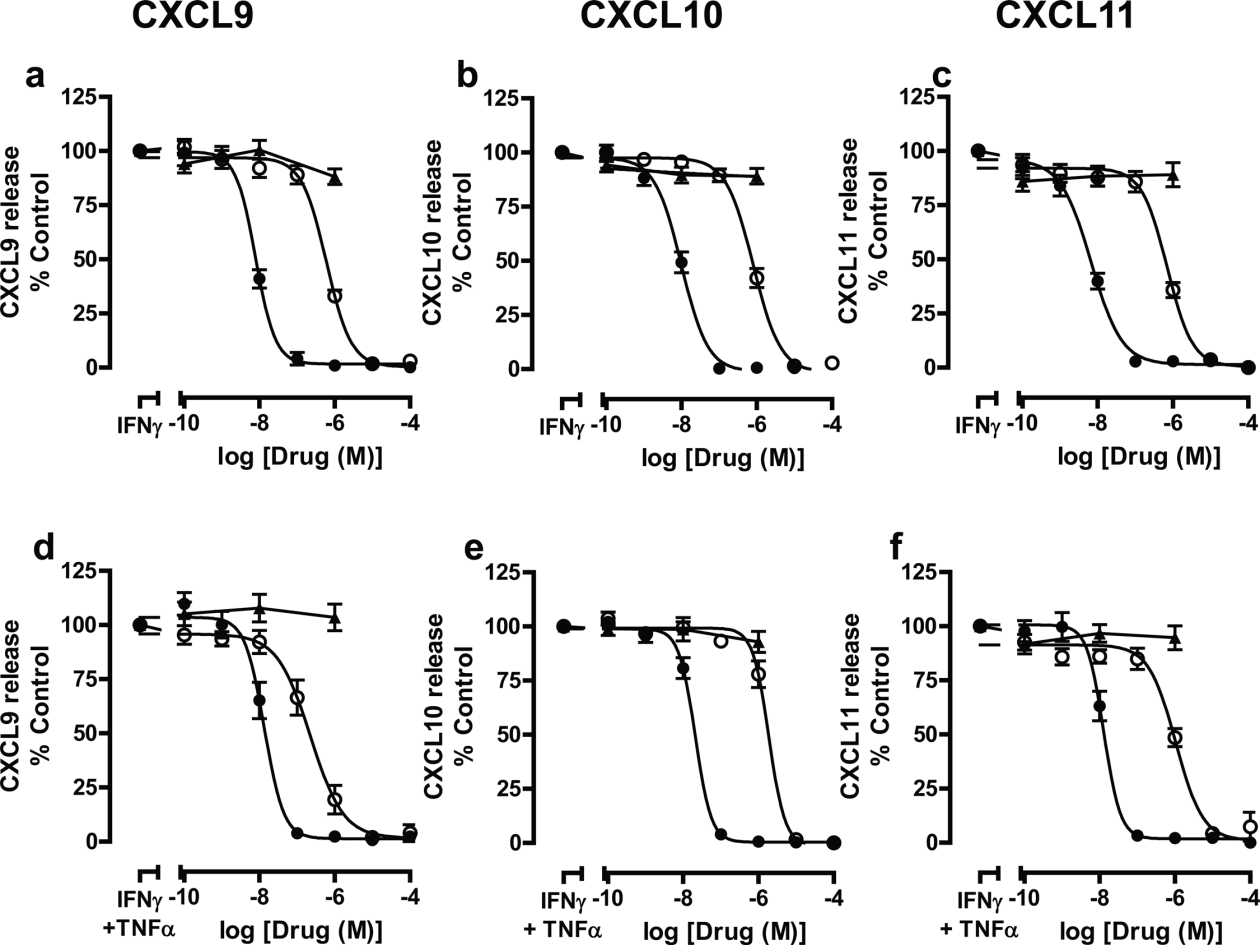
Effect of JAK inhibitors, PF956980 and PF1367550 and dexamethasone on IFNγ and IFNγ+TNFα-stimulated release of CXCL9, CXCL10 and CXCL11 from human primary airway epithelial cells. Human primary airway epithelial cells were pre-treated for 1h with either PF956980 (○), PF1367550 (●), or dexamethasone (▲) followed by stimulation for 20h with IFNγ (10 ng/ml) (panels a-c) or IFNγ + TNFα (10 ng/ml) (panels d-f). Media was harvested and the concentrations of CXCL9 (panels a and d), CXCL10 (panels b and e) and CXCL11 (panels c and f) were measured by ELISA. Data are presented as mean ± SEM, n = 10–13.

It was possible that concomitant addition of stimulus and JAK inhibitors may elicit a different inhibitory profile, therefore to address this, experiments were performed. Addition of both cytokines concomitantly with PF956980 or PF1367550 showed similar inhibitory profiles to those exhibited when inhibitors were added prophylactically (S1 Fig) with no difference in IC_50_ values (S1 Table). Experiments were then performed to determine the effects of both JAK inhibitors in human primary airway epithelial cells in order to determine whether their ability to suppress CXCR3 chemokine release from BEAS-2B cells was reflective of their efficacy in primary cells. Again, both PF956980 and PF1367550, inhibited both IFNγ-stimulated and IFNγ + TNFα-stimulated CXCR3 chemokine release from human primary airway epithelial cells in a concentration-dependent manner irrespective of the source of the primary airway epithelial cells (S2–S4 Figs, Table 4) with cells from non-smokers, smokers and COPD patients responding similarly. Therefore, the data from all primary cells were pooled to increase statistical power (Fig 3) (Table 5) and showed that PF1367550 was ~80-fold more potent than PF956980. In these experimental systems, dexamethasone did not suppress CXCR3 chemokine release.

**Table 4:** IC_50_ values for PF956980 and PF1367550 on IFNγ and IFNγ+TNFα-stimulated release of CXCL9, CXCL10 and CXCL11 from human primary airway epithelial cells from non-smokers, smokers and COPD patients.

	PF956980 EC_50_ (μM)			PF1367550 EC_50_ (nM)		
IFNγ	CXCL9	CXCL10	CXCL11	CXCL9	CXCL10	CXCL11
**Non-smokers**	0.88 ± 0.39 n = 4	0.81 ± 0.15 n = 4	0.66 ± 0.24 n = 4	9.73 ± 3.5 n = 3	12.8 ± 5.6 n = 3	8.5 ± 0.7 n = 3
**Smokers**	0.55 ± 0.09 n = 4	1.28± 0.36 n = 4	0.78 ± 0.16 n = 4	9.0 ± 3.00 n = 4	12.3± 0.36 n = 4	6.8 ± 1.9 n = 4
**COPD**	0.63 ± 0.09 n = 5	0.53 ± 0.01 n = 5	0.82 ± 0.25 n = 5	7.4 ± 1.3 n = 6	7.1 ± 1.3 n = 6	6.3 ± 1.4 n = 6
**IFNγ +TNFα**						
**Non-smokers**	0.61 ± 0.26 n = 4	2.72 ± 0.36 n = 4	1.85 ± 0.67 n = 4	14.1 ± 7.1 n = 3	20.3 ± 5.1 n = 3	13.7 ± 6.7 n = 3
**Smokers**	0.35 ± 0.26 n = 4	1.88 ± 0.41 n = 4	0.87 ± 0.13 n = 4	15.3 ± 4.8 n = 4	20.2 ± 3.6 n = 4	11.8 ± 2.9 n = 4
**COPD**	0.59± 0.38 n = 5	1.84 ± 0.40 n = 5	1.92 ± 0.67 n = 5	20.8 ± 4.8 n = 6	29.1± 3.5 n = 6	16.7 ± 4.1 n = 6

Human primary airway epithelial cells were pre-treated for 1h in the presence of either PF956980 or PF1367550 prior to stimulation for 20h in the absence or presence of IFNγ (10 ng/ml) or IFNγ+TNFα (10 ng/ml). After this time, media was harvested and the concentrations of CXCL9, CXCL10 and CXCL11 measured by ELISA. Data are presented as mean±SEM.

**Table 5:** IC_50_ values for PF956980 and PF1367550 on IFNγ and IFNγ+TNFα-stimulated release of CXCR3 chemokinesCXCL9, CXCL10 and CXCL11 from human primary airway epithelial cells.

	PF956980 IC_50_ (μM)		PF1367550 IC_50_ (nM)	
Chemokine	IFNγ	IFNγ+TNFα	IFNγ	IFNγ+TNFα
**CXCL9**	0.68 ± 0.12	0.52 ± 0.17	8.4 ± 1.3	17.6 ± 3.0
**CXCL10**	0.96 ± 0.19	2.12 ± 0.24	10.4 ± 1.7	24.3 ± 2.4
**CXCL11**	0.76 ± 0.12	1.58 ± 0.33	7.0 ± 0.9	14.5 ± 2.4

Human primary airway epithelial cells were pre-treated for 1h in the presence of either PF956980 or PF1367550 prior to stimulation for 20h in the absence or presence of IFNγ (10ng/ml) or IFNγ+TNFα (10ng/ml). After this time, media was harvested and the concentrations of CXCL9, CXCL10 and CXCL11 measured by ELISA. Data are presented as mean±SEM, n = 13.

In order to determine whether any of the inhibitory effects of the JAK inhibitors could be attributed to cell death, MTT assays were performed at the end of every experiment. PF956980 had no effect on cell viability at any of the concentrations used in this study. PF1367550 had no effect on cell viability within 10^3^ of the IC_50_ for this compound and therefore any observed inhibitory effects are driven the pharmacological mechanism, since at 10^-6^M, and at all concentrations below this, both inhibitors showed no alterations in cell viability compared to untreated cells. Therefore, the effect of the JAK inhibitors to suppress CXCR3 chemokine release is not related to cell toxicity. The limited effect of dexamethasone in these systems suggested that the cells used in this study may be glucocorticosteroid-insensitive. In order to address this issue, the levels of IL-6 released from cells stimulated with IFNγ + TNFα were measured. At baseline, BEAS-2B cells and human primary airway epithelial cells release 41±15 pg/ml, n = 8 and 236±86 pg/ml, n = 7, IL-6 respectively. These levels are increased in the presence of IFNγ + TNFα to 1480±142 pg/ml, n = 4 and 1077±267 pg/ml, n = 7 for BEAS-2B cells and primary airway epithelial cells, respectively. In the presence of dexamethasone, IL-6 release was suppressed by ~50–70% with IC_50_ values of 16±8 nM and 11±7 nM respectively. Therefore, these cells are sensitive to glucocorticosteroid inhibition; however, the release of CXCL9, CXCL10 and CXCL11 following stimulation with either IFNγ or IFNγ in combination with TNFα is not. Both JAK inhibitors also suppressed this response in primary airway epithelial cells but with IC_50_ values of ~5 and 50-fold greater than those for inhibition of CXCR3 chemokine release (PF956980: 11.4 ± 9 μM and PF1367550: 0.8 ± 0.2 μM, n = 7) suggesting that these effects may be unrelated to JAK inhibition directly.

### Effects of JAK inhibitors on STAT-1

**Fig 4:**
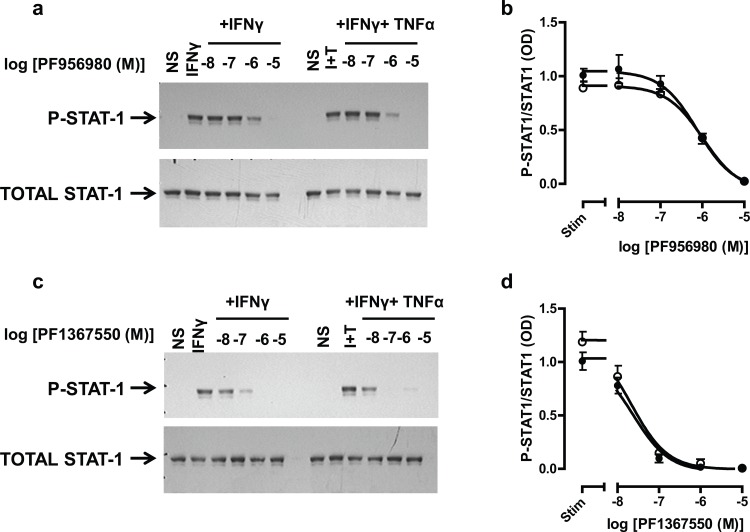
Effect of JAK inhibitors, PF956980 and PF1367550 on IFNγ and IFNγ + TNFα-stimulated phosphorylation of STAT-1 in BEAS-2B cells. BEAS-2B cells were pre-treated for 1h with either PF956980 or PF1367550 followed by stimulation for 10 min with IFNγ (10ng/ml) (○) or IFNγ + TNFα (●). Cells were harvested and western blots performed for phosphorylated STAT-1 and total STAT-1 and quantified by densitometry. Data are representative of n = 4 independent experiments (panels a and c) and presented as mean±SEM, n = 4 (panels b and d).

**Fig 5:**
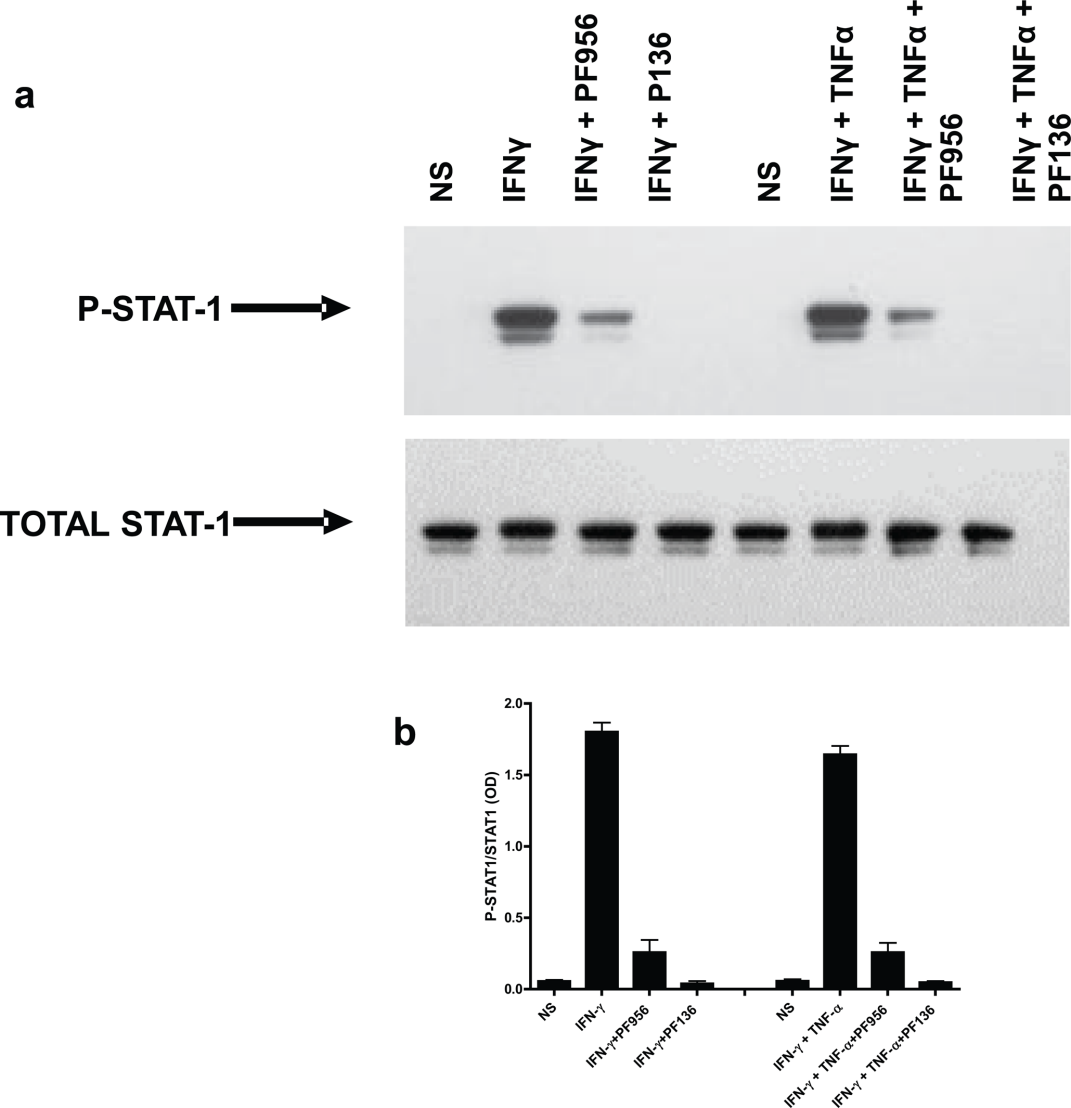
Effect of JAK inhibitors, PF956980 and PF1367550 on IFNγ and IFNγ + TNFα-stimulated phosphorylation of STAT-1 in human primary airway epithelial cells. Human primary airway epithelial cells were pre-treated for 1h with either 1μM PF956980 (PF956) or 1μM PF1367550 (PF136) followed by stimulation for 10 min with either IFNγ (10ng/ml) or IFNγ + TNFα. Cells were harvested and western blots performed for phosphorylated STAT-1 and total STAT-1 and quantified by densitometry. Data are representative of n = 4 independent experiments (panel a) and presented as mean ± SEM, n = 4 (panel b).

**Fig 6:**
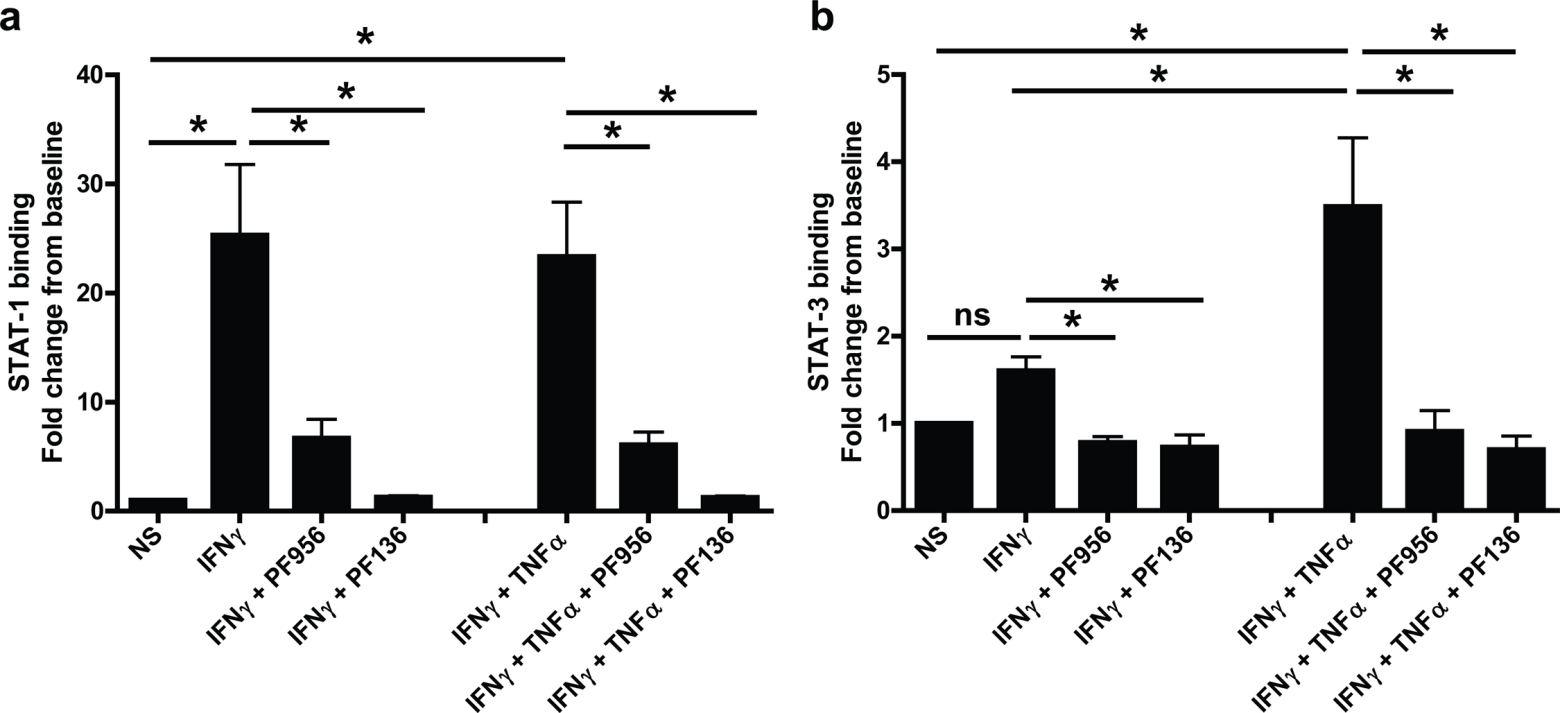
Effect of JAK inhibitors, PF956980 and PF1367550 on IFNγ and IFNγ + TNFα-stimulated DNA binding of STAT-1 and STAT-3 in BEAS-2B cells. BEAS-2B cells were pre-treated with either PF956980 (PF956) or PF1367550 (PF136) for 1h followed by stimulation with IFNγ (10ng/ml) or IFNγ + TNFα. After 1 h, nuclear extracts of the cells were prepared and TransAM assays for STAT-1 (panel a) and STAT-3 (panel b) were performed. Data are presented as fold change from baseline ± SEM; n = 4.

Having ascertained that JAK inhibitors could suppress the glucocorticosteroid-insensitive release of CXCL9, CXCL10 and CXCL11 from airway epithelial cells, it was important to confirm that this was via inhibition of the JAK-STAT pathway and not an off-target effect of these agents. Activation of the JAK-STAT pathway by IFNγ leads to the phosphorylation of STAT-1, therefore experiments were devised to assess whether PF956980 and PF1367550 altered STAT-1 phosphorylation. BEAS-2B cells were stimulated with either IFNγ or IFNγ in combination with TNFα and the phosphorylation of STAT-1 was measured by western blotting as reported previously [15] (Fig 4). Exposure of cells to either IFNγ or IFNγ + TNFα increase STAT-1 phosphorylation and this was inhibited by pre-treatment with either PF956980 or PF1367550 (Fig 4) (IC_50_ values: PF956980; 0.94±0.16μM, and 0.84±0.21μM, n = 4 for IFNγ and IFNγ + TNFα stimulation respectively; PF1367550; 24±6nM, and 32±13nM, n = 4 for IFNγ and IFNγ + TNFα stimulation respectively) with, PF1367550 being ~40-fold more potent at suppressing STAT-1 phosphorylation when compared with PF956980 (Fig 4). These data in BEAS-2B cells were further validated using human primary airway epithelial cells. Both PF956980 and PF1367550 suppressed phosphorylation of STAT-1 by IFNγ and IFNγ + TNFα (Fig 5). The capacity of PF956980 and PF1367550 to suppress JAK-STAT activation was further supported by the observation that both IFNγ and IFNγ + TNFα stimulation of STAT-1 DNA binding could be suppressed by these inhibitors in BEAS-2B cells (Fig 6A). Although, IFNγ failed to promote STAT-3 DNA binding (Fig 6B), IFNγ + TNFα significantly increased STAT-3 binding to DNA in a manner that was suppressed by both PF956980 and PF1367550 (10^-6^M). There was no activation of STAT-5A or STAT-5B in this system (data not shown).

### Effects of JAK inhibitors on *CXCL9*, *CXCL10*, *CXCL11* gene transcription

**Fig 7:**
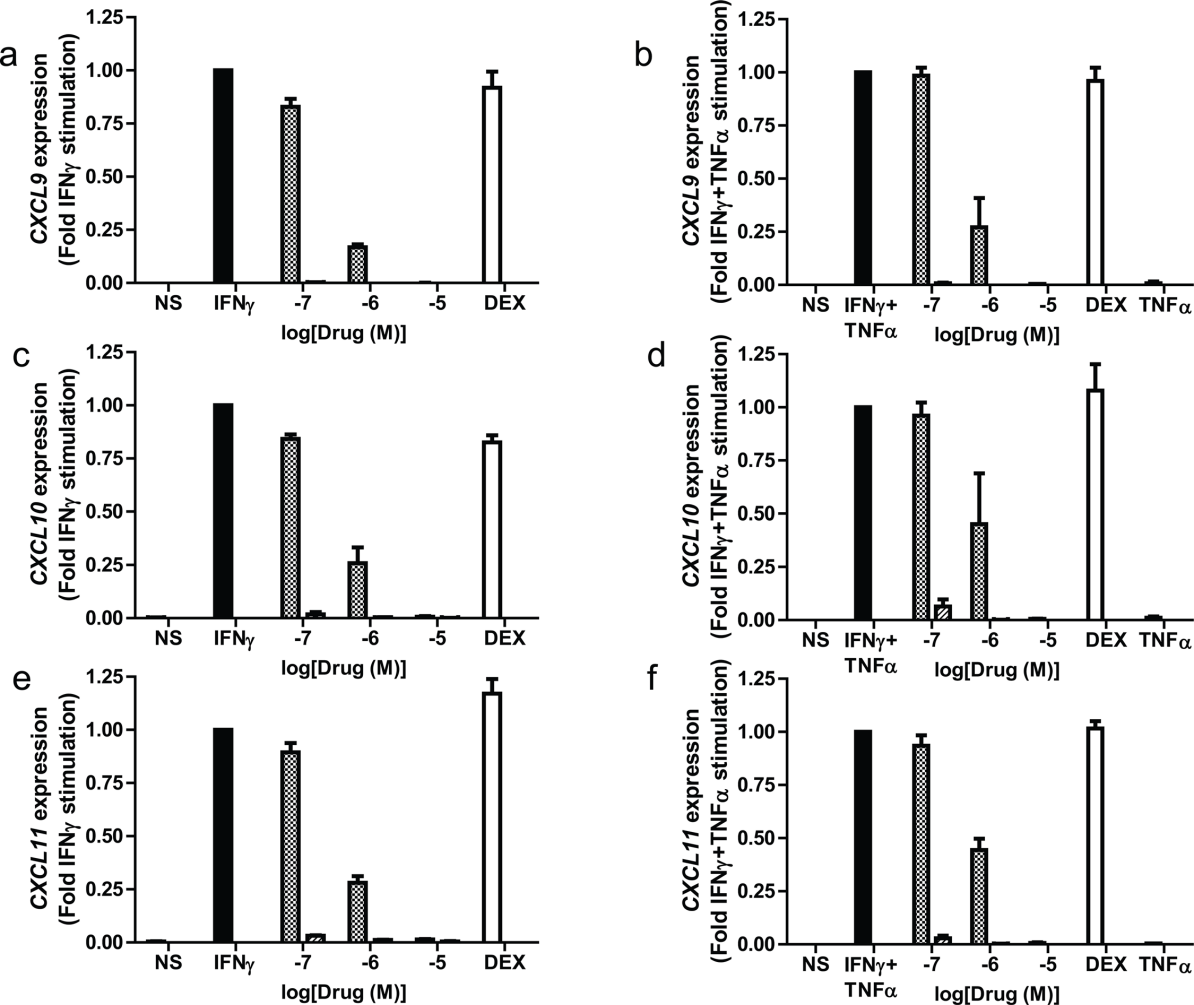
Effect of JAK inhibitors, PF956980 and PF1367550 on IFNγ and IFNγ + TNFα-stimulated expression of *CXCL9*, *CXCL10*, and *CXCL11*. BEAS-2B cells were pre-treated with either PF956980 (checked bars), PF1367550 (hashed bars) or dexamethasone (10^-6^M white bar) for 1h followed by stimulation with IFNγ (10ng/ml) or IFNγ + TNFα for 6 h. Gene expression of *CXCL9* (panels a and b), *CXCL10* (panels c and d) and *CXCL11* (panels e and f) were quantified by Taqman RT-PCR. Data are presented as fold change form stimulus ± SEM; n = 3 where 1 indicates the level of mRNA induced by IFNγ or IFNγ + TNFα in the absence of inhibitors.

In order to demonstrate that suppression of STAT-1 activation leads to reduced gene expression, experiments were performed using BEAS-2b cells that had been pretreated with JAK inhibitors for 1h prior to stimulation with either IFNγ or IFNγ with TNFα for 4h and RNA isolated (Fig 7). PF956980 suppressed expression of *CXCL9*, *CXCL10* and *CXCL11* in a concentration dependent manner irrespective of stimuli (Fig 7). In contrast, PF1367550 completely inhibited expression of all three genes at all concentrations used. Taken together these data suggest that JAK inhibition suppresses release of CXCL9, CXCL10 and CXCL11 by reducing gene transcription which contrasts with the effect of dexamethasone, which had no effect on gene transcription in this system.

## Discussion

Transgenic mice that over-express IFNγ exhibit many of the emphysematous changes observed in COPD [28], thus suggesting that IFNγ is an important molecule driving the development and continuing pathophysiology of this disease. IFNγ is produced by CD8^+^ T lymphocytes and these cells specifically accumulate in the lungs of patients with COPD at the sites of small airways disease [3, 29] and are associated with increased expression of the chemokine receptor, CXCR3 [2]. This suggests that recruitment of these cells is via CXCR3 and that attenuation of recruitment of CD8^+^ cells into the airways of these patients could provide an anti-inflammatory treatment for this glucocorticosteroid-insensitive disease. Since the chemokines that bind to and activate CXCR3 are induced by IFNγ, the present study investigated the effect of inhibiting IFNγ signalling in airway epithelial cells using, for the first time in these cells, selective JAK inhibitors and demonstrated reductions in CXCR3 chemokine release. In addition, the synergistic action of TNFα with IFNγ to increase expression and release of CXCR3 chemokines has been reported previously [14, 15, 21] and since TNFα is also elevated in the airways of patients with COPD [22], the effects of JAK inhibition on this stimulus was also evaluated and showed similar attenuation of chemokine output. These data would suggest that JAK inhibition could lead to a reduction in the accumulation of CD8+ lymphocytes in COPD, however other chemokines and receptors, possibly CCR5 which is also expressed on these cells [3, 30] could provide an alternative avenue for cell migration and activation. It is of note that in diseases such as COPD, increased levels of proteases including neutrophil elastase and matrix metaloproteinases (MMP) such as MMP-2 and MMP-9 [31, 32], could contribute to chemokine processing. There is evidence that CXCL9, CXCL10 and CXCL11 can all be modified in this way leading to possible alterations in function [33].

PF956980 has been shown to be a selective inhibitor of JAK activation, most prominently against JAK2 and JAK3 with no effect on ~30 other kinase pathways including AKT, GSK3β, IKKβ, IKKi, MAPK1, MAPKAP-K2, MASK, MET, and ROCK1 but with modest effects against PKA and Lck at concentrations >5μM [24]. In the present study, this compound completely attenuated IFNγ-stimulated release of CXCL9, CXCL10 and CXCL11. The IC_50_ reported for PF956980 against JAK3 is ~4nM [24], however this compound has been reported to be effective against IL-4-induced cytotoxic resistance in chronic lymphocytic leukaemia cells at concentrations of ~0.3μM [34] in agreement with the potency of PF956980 in the present study. Moreover a second, structurally dissimilar molecule, PF1367550 had a similar profile of activity to PF956980, suggesting that this is not a chemotype-dependent profile, but a function of JAK enzyme inhibition. However, PF1367550 is a more potent molecule in these assays.

Of note, the human airway epithelial cell line, BEAS-2B responded to both stimuli and inhibitors in a similar manner to isolated human primary airway epithelial cells, thus confirming our previous work and that of others that BEAS-2B are a good model for primary cells in this system [14, 15]. The inhibitory effects of both JAK-STAT inhibitors were also observed when TNFα was included with IFNγ to stimulate release of CXCL9, CXCL10, and CXCL11. The synergistic effect of these cytokines to generate CXCL9, CXCL10 and CXCL11 has been reported previously [14, 21] and is thought to be mediated via the action of not only STAT-1 but also NF-κB and CREB [21]. However, inhibition of the JAK pathway continued to inhibit expression and production of CXCL9, CXCL10 and CXCL11 in the presence of TNFα, indicating that inhibition of STAT-1 phosphorylation is an essential regulatory step in this process and indicates that JAK inhibition may be a good therapeutic approach in COPD where multiple inflammatory mediators interact.

Although IFNγ classically signals through STAT-1 homodimers [35], expression of CXCL11 has also been reported to occur via STAT-3 [36]. The present study was unable to measure a significant increase in STAT-3 DNA binding in response to IFNγ stimulation, in contrast to STAT-1. However, basal DNA binding of STAT-3 was attenuated by both JAK inhibitors. STAT-3 is activated during transformation of cells [37] and since BEAS-2B are a transformed cell line [38], STAT-3 is most likely to be activated basally and can then be inhibited by the JAK inhibitors. Nevertheless, DNA binding of STAT3 increased significantly following stimulation of BEAS-2B cells with the combination of IFNγ and TNFα and again could be suppressed by JAK inhibition. This suppression of STAT signalling by the JAK inhibitors used in this study led to inhibition of gene transcription with again PF1367550 being more effective than PF956980. Taken together, these data indicate that suppression of JAK activation by selective, small molecule inhibitors, such as PF1367550, would be of benefit in inflammatory diseases where recruitment of cells via CXCL9, CXCL10 and CXCL11 is important. Currently, pan-JAK inhibitors are undergoing clinical trials in inflammatory diseases including psoriasis and rheumatoid arthritis [39, 40] with Tofacitinib, which is structurally similar to PF956980 [24] already approved for use in rheumatoid arthritis in the USA. As such, a similar approach could be promising in diseases such as COPD where glucocorticosteroids are not effective.

## Supporting Information

S1 Fig Effect of JAK inhibitors, PF956980 and PF136550 and dexamethasone on IFNγ and IFNγ + TNFα-stimulated release of CXCL9, CXCL10 and CXCL11 from BEAS-2B cells when added concomitantly. BEAS-2B cells were incubated for with either PF956980 (○), PF1367550 (●), or dexamethasone (▲) and stimulated for 20h with IFNγ (10ng/ml) (panels a-c) or IFNγ + TNFα (10ng/ml) (panels d-f). Media was harvested and the concentrations of CXCL9 (panels a and d), CXCL10 (panels b and e) and CXCL11 (panels c and f) were measured by ELISA. Data are presented as mean±SEM, n = 7–8.
(EPS)

S2 Fig Effect of JAK inhibitors, PF956980 and PF1367550 and dexamethasone on IFNγ and IFNγ + TNFα-stimulated release of CXCL9 from human primary airway epithelial cells derived from non-smokers, smokers and patients with COPD. Human primary airway epithelial cells from non-smokers (panels a and d), smokers (panels b and e) and patients with COPD (panels c and f) were pre-treated and incubated for 1h with either PF956980 (○), PF1367550 (●), or dexamethasone (▲) followed by stimulation for 20h with either IFNγ (10ng/ml) (panels a-c) or IFNγ + TNFα (10ng/ml) (panels d-f). Media was harvested and the concentrations of CXCL9 were measured by ELISA. Data are presented as mean±SEM, n = 5–6.
(EPS)

S3 Fig Effect of JAK inhibitors, PF956980 and PF1367550 and dexamethasone on IFNγ and IFNγ + TNFα-stimulated release of CXCL10 from human primary airway epithelial cells derived from non-smokers, smokers and patients with COPD. Human primary airway epithelial cells from non-smokers (panels a and d), smokers (panels b and e) and patients with COPD (panels c and f) were pre-treated and incubated for 30 min with either PF956980 (○), PF1367550 (●), or dexamethasone (▲) followed by stimulation for 20h with either IFNγ (10ng/ml) (panels a-c) or IFNγ + TNFα (10ng/ml) (panels d-f). Media was harvested and the concentrations of CXCL10 were measured by ELISA. Data are presented as mean±SEM, n = 5–6.
(EPS)

S4 Fig Effect of JAK inhibitors, PF956980 and PF1367550 and dexamethasone on IFNγ and IFNγ + TNFα-stimulated release of CXCL11 from human primary airway epithelial cells derived from non-smokers, smokers and patients with COPD. Human primary airway epithelial cells from non-smokers (panels a and d), smokers (panels b and e) and patients with COPD (panels c and f) were pre-treated and incubated for 30 min with either PF956980 (○), PF1367550 (●), or dexamethasone (▲) followed by stimulation for 20h with either IFNγ (10ng/ml) (panels a-c) or IFNγ + TNFα (10ng/ml) (panels d-f). Media was harvested and the concentrations of CXCL10 were measured by ELISA. Data are presented as mean±SEM, n = 5–6.
(EPS)

S1 Table IC_50_ values for PF956980 and PF1367550 when incubated concomitantly with IFNγ and IFNγ+TNFα for the release of CXCL9, CXCL10 and CXCL11 from BEAS-2B cells. BEAS-2B cells were incubated with either PF956980 or PF1367550 for 20h in the absence or presence of IFNγ (10 ng/ml) or IFNγ+TNFα (10 ng/ml). After this time, media was harvested and the concentrations of CXCR3 chemokines measured by ELISA. Data are presented as mean±SEM, n = 4
(DOCX)
