# Supplementary material for: Effect of JAK Inhibitors on Release of CXCL9, CXCL10 and CXCL11 from Human Airway Epithelial Cells
Source: PLoS One. 2015 Jun 19;10(6):e0128757. doi: 10.1371/journal.pone.0128757 (PMC4474874; doi:10.1371/journal.pone.0128757)
Supplement: S1 Table — BEAS-2B cells were incubated with either PF956980 or PF1367550 for 20h in the absence or presence of IFNγ (10 ng/ml) or IFNγ+TNFα (10 ng/ml). After this time, media was harvested and the concentrations of CXCR3 chemokines measured by ELISA. Data are presented as mean±SEM, n = 4 (DOCX) [file pone.0128757.s005.docx]

|  | **PF956980**  **IC_50_ (µM)** | | **PF1367550**  **IC_50_ (nM)** | |
| --- | --- | --- | --- | --- |
| **Chemokine** | **IFNγ** | **IFNγ+TNFα** | **IFNγ** | **IFNγ+TNFα** |
| **CXCL9** | 0.3 ± 0.1 | 1.0 ± 0.3 | 10.3 ± 2.1 | 31.6 ± 13.4 |
| **CXCL10** | 1.1 ± 0.4 | 4.0 ± 1.3 | 16.6 ± 2.7 | 146.1 ± 32.6 |
| **CXCL11** | 1.1 ± 0.3 | 1.1 ± 0. 3 | 16.0± 4.1 | 32.0 ± 8.7 |

**Table S1. IC_50_ values for PF956980 and PF1367550 when incubated concomitantly with IFNγ and IFNγ+TNFα for the release of ELR negative CXCR3 chemokines from BEAS-2B cells.**

BEAS-2B cells were incubated with either PF956980 or PF1367550 for 20h in the absence or presence of IFNγ (10 ng/ml) or IFNγ+TNFα (10 ng/ml). After this time, media was harvested and the concentrations of CXCR3 chemokines measured by ELISA. Data are presented as mean±SEM, n=4
